# Pressure Sensor Placement for Leak Localization in Water Distribution Networks Using Information Theory

**DOI:** 10.3390/s22020443

**Published:** 2022-01-07

**Authors:** Ildeberto Santos-Ruiz, Francisco-Ronay López-Estrada, Vicenç Puig, Guillermo Valencia-Palomo, Héctor-Ricardo Hernández

**Affiliations:** 1 TURIX-Dynamics Diagnosis and Control Group, Tecnológico Nacional de México, I.T. Tuxtla Gutiérrez, Carretera Panamericana km 1080 S/N, Tuxtla Gutierrez 29050, Mexico; ildeberto.dr@tuxtla.tecnm.mx (I.S.-R.); hector.hl@tuxtla.tecnm.mx (H.-R.H.); 2 Institut de Robòtica i Informàtica Industrial, CSIC-UPC, Universitat Politècnica de Catalunya, C/. Llorens i Artigas 4-6, 08028 Barcelona, Spain; vicenc.puig@upc.edu; 3 Tecnológico Nacional de México, I.T. Hermosillo, Av. Tecnológico y Periférico Poniente S/N, Hermosillo 83170, Mexico; gvalencia@hermosillo.tecnm.mx

**Keywords:** sensor placement, pressure monitoring, information theory, leak localization, water distribution network

## Abstract

This paper presents a method for optimal pressure sensor placement in water distribution networks using information theory. The criterion for selecting the network nodes where to place the pressure sensors was that they provide the most useful information for locating leaks in the network. Considering that the node pressures measured by the sensors can be correlated (mutual information), a subset of sensor nodes in the network was chosen. The relevance of information was maximized, and information redundancy was minimized simultaneously. The selection of the nodes where to place the sensors was performed on datasets of pressure changes caused by multiple leak scenarios, which were synthetically generated by simulation using the EPANET software application. In order to select the optimal subset of nodes, the candidate nodes were ranked using a heuristic algorithm with quadratic computational cost, which made it time-efficient compared to other sensor placement algorithms. The sensor placement algorithm was implemented in MATLAB and tested on the Hanoi network. It was verified by exhaustive analysis that the selected nodes were the best combination to place the sensors and detect leaks.

## 1. Introduction

Finding a suitable sensor placement is a fundamental problem for monitoring water distribution networks (WDNs) because it is impossible to install sensors at each point of the geographic area covered by the distribution system. A WDN comprises hundreds of nodes; however, only a few sensors can be installed in certain carefully selected nodes. Then, the main question is how to select the optimal sensor placement. Finding an answer to this problem is not trivial because the selected nodes must capture the most relevant information to estimate hydraulic variables at non-measured points and provide essential information for different supervision algorithms, e.g., for leak localization [1,2]. Often there are pressure and flow instruments at the supplying nodes of a WDN and in some cases at critical points (e.g., at the minimum pressure node). However, these measurements are not sufficient for an accurate leak localization, so additional sensors must be installed at other sites [3]. A practical solution is to install more pressure sensors, because they are cheaper and easier to install and maintain than flow sensors. In addition, node pressures are more sensitive to leaks than flow rates, which is why many localization algorithms are based primarily on pressure measurements. The problem of sensor placement is closely related to other WDN management problems, such as the state estimation of the network [4,5,6], model calibration [7,8], water quality monitoring such as detection of contaminants and cyberattacks [9,10,11,12,13,14,15], among others. Nevertheless, the present work focuses on the context of leak detection and localization as discussed in [16,17]. Regarding techniques for optimal sensor placement for leak/burst detection and localization in water distribution systems, a comprehensive review can be found at [18].

In a mathematical/computational context, the placement of pressure sensors is a mixed-integer programming problem. In this problem, for a network with *N* nodes, a sensor placement consists of a selection $\left[s_{1},s_{2},\ldots ,s_{N}\right]$ where $s_{i}$ are binary decision variables such that $s_{i}=1$ indicates that a sensor will be placed on the *i*-th node, whereas $s_{i}=0$ indicates that no sensor will be placed on that node.

Combinatorial analysis shows that there are $2^{N}-1$ possible sensor placements when non-empty subsets with any number of sensors are considered. If the number of sensors is previously set to a fixed number *S*, then the number of possible sensor placements is reduced to NS, which is still a very large number. Therefore, in medium-sized and large networks, it is not feasible to check all possible combinations. For example, in a network containing 500 nodes the number of different placements for 10 sensors is 50010≈2.5 × 1020. That is why it is important to find an optimal placement method without analyzing all the possible combinations.

Usually, sensor placement focused on leak localization is addressed with an optimization approach from synthetic pressure data obtained by simulation. Some authors have focused on minimizing the number of undetectable leaks [19,20], whereas others reduce the error in the leak location [16,21]. In [22], a min-max optimization algorithm that considers the isolation of the leaks from their signatures obtained through simulation is proposed. In [23], a multi-objective approach to mitigate errors both in the detection and localization of leaks, considering minimum night flow conditions, is presented. Regarding the optimization of the objective function, two approaches are usually used: deterministic methods (e.g., branch and bound [24]) and metaheuristic methods, (e.g., genetic algorithms [25,26,27] and particle swarm optimization [28]). Deterministic approaches guarantee an optimal solution, but the computation time increases exponentially with the number of nodes and possible leak scenarios. On the other hand, metaheuristic methods search for a near-optimal solution that only guarantees optimality when the number of candidate solutions evaluated (named “population size”) tends to infinity. Furthermore, optimization-based sensor placement methods are linked to a specific leak localization method because the objective function is expressed in terms of a localization error or isolation index for that method [16,28,29]. Based on this, a sensor placement method may be optimal for one specific leak localization method but not as good for others. Furthermore, the method should be independent of the leak localization method since it is not feasible to change it for every method. Thus, an improved leak localization method could be proposed based on an ensemble of different machine learning algorithms using the information provided by the sensors.

The huge computing time in networks with hundreds and thousands of nodes using optimization-based methods and the high dependence on the selected leak localization method has motivated the present work. In this new proposal, it is not considered how specific leak localization methods will use the information provided by the sensors, but rather that the sensor placement method only focuses on the sensors capturing as much information related to the leaks as possible. The proposed method consists of a heuristic algorithm to select the subset of nodes where to place the sensors, seeking to maximize the relevance of the information captured by the sensors while minimizing the redundancy between the pressures in the selected nodes. Both metrics, relevance and redundancy, are defined in terms of information theory.

An important contribution of this work is the reduction in computing time for sensor placement, compared to methods based on metaheuristic optimization. Another relevant contribution is the nondependence of the sensor placement on the leak localization method used, which allows the use of the same sensor placement with different localization methods. Some aspects not yet covered in this work are the possible heterogeneity of the sensors (e.g., different errors and measurement ranges) and the influence of the measurement noise in the optimal placement, but they are considered as future work.

The rest of the document is organized as follows: in Section 2, the concepts of redundancy and relevance is presented in terms of mutual information, and the information quotient used as the basis of the method is also defined. In Section 3, the proposed method is formally described and some guidelines for its implementation are given. In Section 4, the results of the proposed method applied to a simplified version of the Hanoi network (case study) are presented. Finally, in Section 5, the conclusions are presented and future related works are proposed.

## 2. Information Theory Fundamentals

In Shannon’s information theory (IT), the self-information of a random variable is defined according to the unexpectedness of its values [30]. Thus, the information contained in a constant random variable is zero. Mathematically, if an event *E* has probability *P*, its information content is defined by:

(1) $$I\left(E\right)\;\overset{\mathrm{def}}{=}\;-\log_{b}\left(P\right),$$

where the unit of measure of *I* is defined by the base of the logarithm, *b*, which is called “bit” if b=2. In a discrete random variable *X* with probability function p(x)=Pr(X=x), the self-information for obtaining *x* as a result when measuring *X* is given by:

(2) $$I\left(x\right)=-\log_{b}\left(p\left(x\right)\right)=\log_{b}\left(1/p\left(x\right)\right).$$

To quantify the average information that a random variable contains, considering all its possible values, the *entropy* is used:

(3) $$H\left(X\right)\;\overset{\mathrm{def}}{=}\;E\left(I\left(x\right)\right)=\sum_{x}-p\left(x\right)\log_{b}\left(p\left(x\right)\right),$$

which is the expected value of the information contained in the measurements of *X*, that is, the sum of the self-information of each of its possible values weighted by its probability of occurrence.

The mutual information of two random variables, sometimes called “information gain”, measures the amount of information obtained from one of the random variables by observing the other one. For example, in a practical application of WDN monitoring, the mutual information between two node pressures would indicate how much information about the pressure at one node is gained by knowing the pressure at the other one. In probabilistic terms, the mutual information determines how different the joint distribution of (X,Y) is from the product of the marginal distributions of *X* and *Y*.

For two discrete variables *X* and *Y*, defined over the space X×Y, the mutual information is computed as the double sum:

(4) $$I\left(X,Y\right)=\sum_{y\in Y}\sum_{x\in X}p\left(x,y\right)\log\frac{p(x,y)}{p(x)p(y)},$$

where p(x,y)=Pr(X=x,Y=y) is the joint probability function of *X* and *Y*, whereas p(x) and p(y) are the marginal probability functions of *X* and *Y*, respectively. The mutual information (4) is derived from the entropy and the conditional probability by the following equivalences:

(5) I(X,Y)≡H(X)−H(X|Y)≡H(Y)−H(Y|X).

Furthermore, I(X,X)=H(X), I(X,Y)=I(Y,X) and I(X,Y)≥0, where I(X,Y)=0 iff *X* and *Y* are independent.

For continuous random variables, the summations in (4) are replaced by integrals and the probability functions by probability densities:

(6) $$I\left(X,Y\right)=\int_{Y}\int_{X}p\left(x,y\right)\log\frac{p(x,y)}{p(x)p(y)}\;dx\;dy.$$

Due to the difficulty in modeling the probability densities and subsequently evaluating the double integrals in (6), a simplification to calculate the mutual information in continuous variables is to discretize the variables with *n* bits, so that the domain of each variable is reduced to $2^{n}$ bins. For example, to compute the mutual information of two node pressures in a hydraulic network, the span of the pressure variables $\left[P_{\min},P_{\max}\right]$ must be divided into a discrete 8-bit grid (256 different values) and then (4) is applied.

## 3. Sensor Placement Method

The proposed sensor placement method is based on a dataset of node pressures that collects typical variations due to leaks of different sizes in all network nodes. The pressure dataset is obtained from simulations with the hydraulic model of the network in [31]. Each pressure data point is labeled with a “leak class” (the node where the leak occurs) so that the proposed method can be classified as supervised.

In the context of machine learning, the placement of pressure sensors is a *feature selection* stage. To select the features (the subset of nodes where the sensors will be placed), an algorithm is proposed that seeks to maximize the relevance of the selected features (node pressures) for the response variable (leaky node), while each of them avoids capturing information already contributed by the others, that is, minimizing redundancy.

The following definitions of relevance and redundancy, proposed in [32], are used as a basis for defining the methodology:

**Definition** **1** (Relevance)**.** *A metric of the relevance of the subset of node pressures S for the response variable y (leak node), is given by*

(7) $$\mathrm{Rel}\left(S\right)\;\overset{\mathrm{def}}{=}\;\frac{1}{S}\sum_{x\in S}I\left(x,y\right),$$

*where x is any feature in S, and $S=\left|S\right|$ is the number of features in S (the cardinality).*

**Definition** **2** (Redundancy)**.** *A metric for information redundancy in a feature subset S is given by:*

(8) $$\mathrm{Red}\left(S\right)\;\overset{\mathrm{def}}{=}\;\frac{1}{S^{2}}\;\;\sum_{x,x^{\prime }\in S}I\left(x,x^{\prime }\right),$$

*where x and $x^{\prime }$ are any features in S.*

To apply the above definitions to compute a pressure sensor placement, first, a dataset of node pressures is built covering different scenarios that consider leaks of different magnitude in all nodes of the network. Through simulation with the hydraulic model of the network, a series of samples of the node pressures is obtained, one sample for each different leakage scenarios. In this way, if *M* different leakage scenarios are simulated in a network containing *N* nodes, the result of the simulation is a collection of *NM*-dimensional vectors, *x* and $x^{\prime }$ in (7) and (8), corresponding to the *N* candidate nodes (initially, it is assumed that all nodes are potential sensing nodes). In addition, an output vector, *y* in (7), is generated containing integer labels to indicate the leaky node corresponding to each simulated scenario.

The exhaustive search for the optimal subset of sensors, S, requires testing the $2^{N}-1$ different combinations, which would require an impractical computation time in networks with many nodes. Therefore, the use of the method proposed in [32] was considered to rank the node pressures through an iterative forward scheme that only requires O(NS) computations. In fact, with this proposal, it is possible to rank all the node pressures in order of importance with a computational cost of $O(N^{2})$.

Next, a heuristic algorithm is proposed, which orders the node pressures according to their importance to explain the different leak classes (leaky nodes). The first node pressures in the output list correspond to the nodes with the highest importance for explaining the leak positions according to the information contained in the dataset. The sequential selection of nodes starts from an empty subset and, at each iteration, adds the best-ranked node among those that are still available to be selected. At each iteration, the relevance of each available feature (node pressure) with respect to the output (leaky node) and its redundancy with respect to the variables that have been previously selected is evaluated using the following equations, adapted from (7) and (8):

(9) $$\begin{array}{cc} {\mathrm{Rel}}_{y}(x) & =I(x,y), \end{array}$$

(10) $$\begin{array}{cc} {\mathrm{Red}}_{S}(x) & =\frac{1}{S}\sum_{x^{\prime }\in \;S}I\left(x,x^{\prime }\right). \end{array}$$

Since maximizing relevance and simultaneously minimizing redundancy represents a multiobjective problem, a combined relevance/redundancy index (RRI) is defined that increases with increasing relevance and also with decreasing redundancy, so the problem is expressed as a single objective to be maximized:

(11) $$\mathrm{RRI}={\mathrm{Rel}}_{y}(x)/{\mathrm{Red}}_{S}(x).$$

The complete node ranking process is formally expressed in Algorithm 1. When the process finishes, the nodes where to place the sensors are taken from the first positions in the list S. If it is not necessary to obtain the complete ranking of the nodes, but only to know the best-ranked positions, the process may stop prematurely when the subset S already contains the number of sensors to be placed.

**Algorithm 1:** Node ranking based on information theory. **Data**:Set with all node pressures, A. The nodes in A will be placed in the ordered list S according to their importance (relevance/redundancy). During theprocess, $\tilde{S}$ denotes the elements of A not yet added in S.**Result**: Set with ordered node pressures, S.
 Initialization: 
$\;S\leftarrow \left[\;\underset{x\in A}{\arg\;\max}\;{\mathrm{Rel}}_{y}\left(x\right)\;\right]$
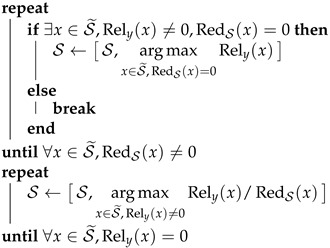
 $S\leftarrow \left[\;S,\tilde{S}\;\right]$


The number of sensors to place for leak localization purposes is determined by the equipment available in most cases. The minimum number of sensors for a successful leak localization method will depend on how that method uses the available information, the measurement noise, as well as the quality, resolution and calibration of the sensors. If there are enough resources to intensively instrument the network, it must be taken into account that increasing the number of sensors does not always lead to better performance in locating leaks. To determine how many sensors should be placed, it is suggested to start from the ranking obtained by Algorithm 1, and run a marginal analysis with the leak localization method to be used. Starting from one sensor (the best ranked), the number of sensors is progressively increased and the leak localization performance is evaluated for each new set of sensors until adding a new sensor no longer represents a significant benefit for locating leaks.

It should be noted that Algorithm 1 does not take into account the geographical distribution of the nodes, since relevance and redundancy depend only on the mutual information between node pressures. This means that the network topology is what determines the amount of mutual information rather than the distance between sensors (i.e., two sensors can be geographically very close but have little mutual information).

## 4. Results and Discussion

**Figure 1:**
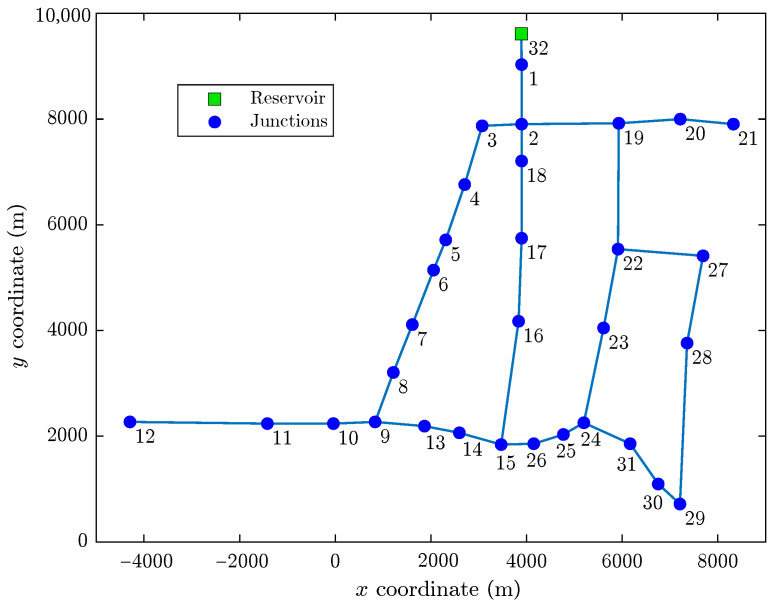
The Hanoi network.

Algorithm 1 was implemented in MATLAB and tested on the Hanoi network [33]. The model of the Hanoi network is composed of one reservoir, 31 consumer nodes, and 34 pipes, as shown in Figure 1. Due to its reduced topology, this network has been used as a standarized benchmark in different works [21,27,34].

In order to build the pressure dataset, leaks of different magnitude were simulated at each junction node using the EPANET 2 simulation program [35] through the EPANET/ MATLAB Toolkit [36]. The procedure to generate the dataset using EPANET, the training and the predictive use of classifiers in locating leaks have been described in [37]. The dataset generated by simulation for this work considered leaks at all junction nodes with flow rates from 50 L/s. In order to simulate leaks at a node, the demand assigned to that node in the EPANET hydraulic model was modified by increasing this demand by an amount equal to the flow of the simulated leak. Because the Hanoi network contains few nodes, the optimality of the sensor placement calculated by Algorithm 1 was exhaustively verified.

To assess the optimality of the sensor placement obtained from Algorithm 1, leak localization tests were carried out using two machine learning methods that used the pressures in the selected nodes as features (input variables). The methods used were the *k*-nearest neighbors (*k*-NN) and quadratic discriminant analysis (QDA). These leak localization methods are based on classifiers that recognize directional patterns in pressure residuals using supervised learning techniques, as described in [38].

Through the marginal analysis, suggested at the end of Section 3, it was determined that S=3 is an adequate number of sensors in the Hanoi network, because the addition of the fourth sensor does not produce a statistically significant improvement (with 0.95 confidence level) in leak location (considering that measurement noise may possibly increase the minimum number of sensors, but this discussion has been considered as future work). Because the Hanoi network contains few nodes, it was possible to comprehensively analyze all 4495 possible combinations of three sensor nodes. For each triplet of nodes (three-sensor placement), 50 leak localization tests were carried out with flow rates $q_{\mathrm{leak}}=1,2,\ldots ,$ 50 L/s at each node of the network. Finally, the overall performance of both methods was evaluated for each candidate triplet using the classification accuracy (Acc) and the average topological distance (ATD) as performance metrics, as defined in [39]. The Acc is the fraction of exactly located leaks considering all leak scenarios in the test dataset, where Acc=1 means that all leaks were correctly located, whereas Acc=0 means that no leaks were correctly located. The ATD is a measure of how far from the true leaky node the classifier locates the leak, counting the number of separation links between the true leaky node and the estimated leaky node, averaged across all scenarios in the test dataset. Therefore, the best sensor placements are the ones that lead to the highest Acc values and the lowest ATD values.

The results in Table 1 show that the node triplet {12,21,28} computed by Algorithm 1 is among the best ranked, since it presents the highest accuracy and the lowest average topological distance.

**Figure 2:**
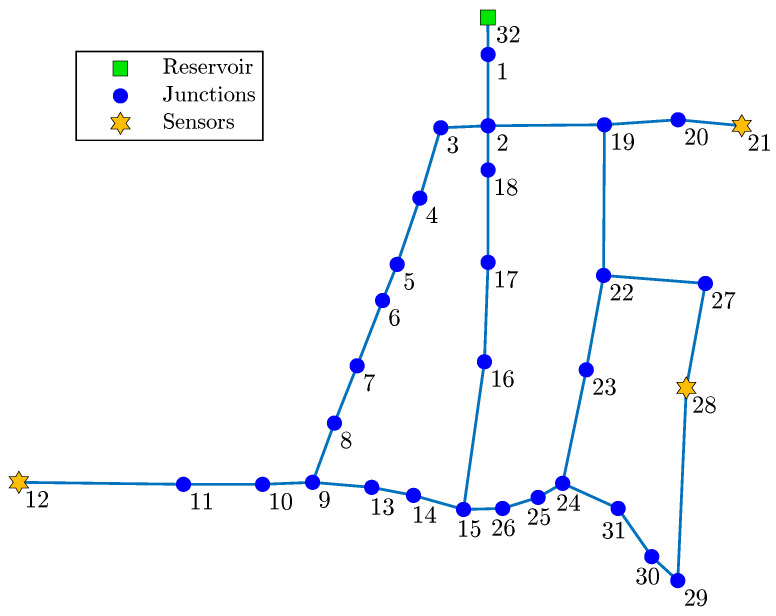
Computed three-sensor placement in the Hanoi network.

**Figure 3:**
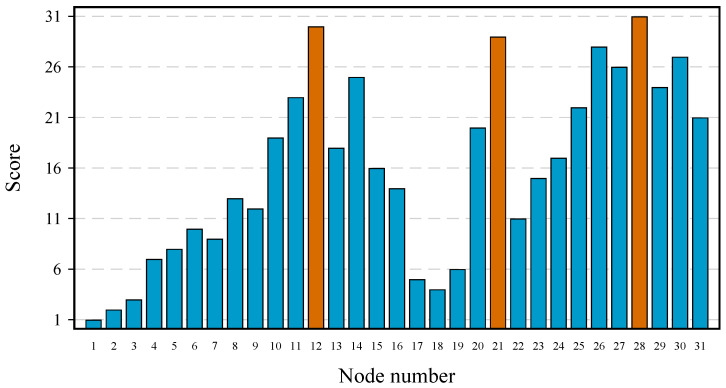
Node ranking in the Hanoi network.

Figure 2 shows the geographic location of the three-sensor placement obtained considering the three nodes best ranked by Algorithm 1. Figure 3 shows the complete ranking considering the 31 nodes of the network.

Table 2 shows the sensor placements obtained for two, three and four sensors in the Hanoi network, and they are compared with the results obtained by metaheuristic methods reported in the literature [28]. The nodes selected by these methods are quite similar and produce very close results in terms of accuracy in locating leaks based on the pressures of the selected nodes. However, there is an important difference in the computation time of the IT-based method (Algorithm 1) compared with the metaheuristic methods. On a personal computer with an Intel 64-bit processor and 8 GB of RAM, the computation time for the IT-based method was around one second with the synthetic data from the Hanoi network, whereas it was 24 min for the genetic algorithm (it may be larger, depending on the initial population size) and about one hour for the exhaustive analysis.

**Figure 4:**
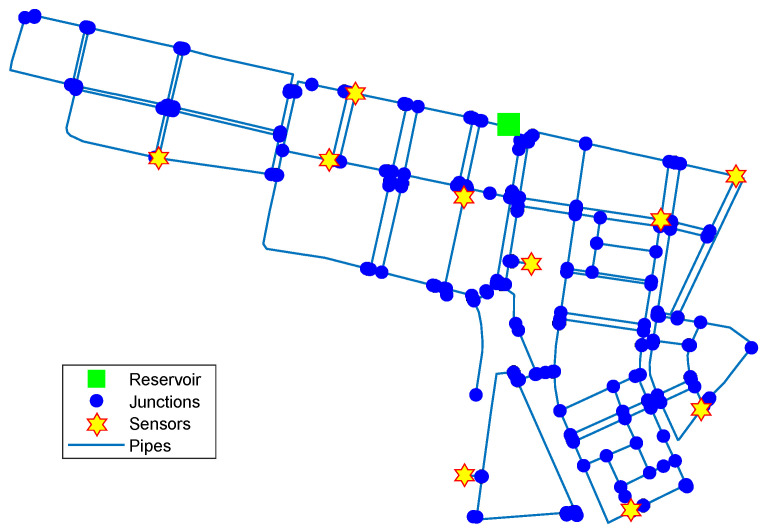
The optimal 10-sensor placement in a sector of the Madrid network.

Further tests were made on larger networks, e.g., in some midsize sectors of the Madrid network. Figure 4 shows a 10-sensor placement obtained using Algorithm 1 in a sector of the Madrid network containing one reservoir, 312 junction nodes and around 14 km of pipes. In this case, optimality was not exhaustively tested due to the vast number of possible placements to compare. However, it was found that the average accuracy in leak localization with sensor placements obtained by Algorithm 1 was at least better than that obtained with an existing placement (previously obtained by a genetic algorithm) for different leak scenarios.

Figure 2 and Figure 4 show that the computed sensor placements do not show geometric regularity (i.e., the sensors do not appear equally spaced), since geometric or spatial criteria are not used to distribute the sensors in the network. However, regardless of geometric irregularity, leak location tests with these placements demonstrated that pressure measurements at these nodes provided the most useful information for discerning between different leak scenarios. In fact, when the placement of sensors obtained by Algorithm 1 is compared with the results reported by other authors using metaheuristics, sometimes very close performances can be found even though the sensors are distributed in different nodes, because the proposed algorithm does not optimize the position of each sensor individually but the entire set of sensors. This can be explained with an informal analogy: two soccer teams can achieve similar performances using different players.

Although, as noted above, there may be different sensor placements that lead to a good performance in locating leaks, the one obtained by Algorithm 1 has the advantage of being calculated in less time than the methods based on metaheuristics and that it is not linked to a specific leak location method, so changing the leak location method does not imply changing the location of the sensors, which would be impractical.

## 5. Conclusions

This paper has presented a technique for finding optimal sensor placements from information theory using a sequential forward selection, maximizing the relevance and minimizing the redundancy of the selected node subset. The proposed technique is computationally less expensive than other methods reported in the literature because the proposed technique operates directly on the values of node pressures without performing calculations for leak localization in the implementation of the algorithm. The optimality of the sensor placement obtained with the proposed method was extensively tested by simulation with the Hanoi network. It was found that the selection of nodes where to place sensors using information theory produced the best combination of pressure variables to locate leaks using different machine learning methods.

An implicit assumption in the proposed algorithms is that all network nodes have the same availability to place the sensors. However, in practice, some specific nodes may have placement priority over others; for example, critical nodes (points of minimum pressure) and nodes that supply essential services (e.g., hospitals) could be monitored as a priority. It may also occur that some nodes already have a sensor installed and that previous partial placement must be held, or that the conditions in a node are physically adverse and instrumentation is avoided. These circumstances warrant adjustments to the proposed sensor placement algorithm that may lead to future work. Another possible working line is the combination of heterogeneous sensors where different sensing specifications are included (e.g., different precision) or where the sensors measure different physical magnitudes (e.g., sensor placements combining pressure and flow sensors).

## Figures and Tables

**Table 1:** Better positions to place three sensors in the Hanoi network, obtained by exhaustive analysis. The shaded selection is the one obtained by Algorithm 1.

**Rank**	**Nodes**	**Location Method**	
		***k*-NN**	**QDA**
1	{12, 21, 28}	0.9974	0.9948
1	{12, 21, 27}	0.9974	0.9948
1	{12, 21, 31}	0.9974	0.9948
2	{7, 12, 21}	0.9961	0.9936
2	{12, 17, 21}	0.9961	0.9936
3	{3, 12, 21}	0.9961	0.9923
3	{4, 12, 21}	0.9961	0.9923
3	{6, 12, 21}	0.9961	0.9923
3	{5, 12, 21}	0.9961	0.9923
(a) Metric: classification accuracy			
**Rank**	**Nodes**	**Location Method**	
		**k-NN**	**QDA**
1	{12,21,28}	0.0026	0.0052
1	{12, 21, 27}	0.0026	0.0052
1	{12, 21, 31}	0.0026	0.0052
2	{12, 13, 21}	0.0065	0.0065
3	{7, 12, 21}	0.0065	0.0090
3	{12, 17, 21}	0.0065	0.0090
4	{3, 12, 21}	0.0039	0.0129
4	{4, 12, 21}	0.0039	0.0129
5	{6, 12, 21}	0.0065	0.0129
(b) Metric: average topological distance			

**Table 2:** Optimal three-sensor placement in the Hanoi network using different methods.

*S*	IT ^*a*^	GA ^*b*^	PSO ^*c*^	SE ^*d*^
2	{12,28}	{12,21}	{12,21}	{12,21}
3	{12,21,28}	{12,21,27}	{12,14,21}	{12,21,29}
4	{12,21,26,28}	{1,12,21,29}	{1,12,21,24}	{1,12,21,29}

^*a*^ Algorithm 1. ^*b*^ Genetic algorithm, reported in [28]. ^*c*^ Particle swarm optimization, reported in [28]. ^*d*^ Semi-exhaustive search, reported in [28].
